# A phantom human chorionic gonadotropin in the case of molar pregnancy

**DOI:** 10.1093/omcr/omae038

**Published:** 2024-05-20

**Authors:** Hirokazu Usui, Asuka Sato, Eri Katayama, Natsuko Nakamura, Kaori Koga

**Affiliations:** Department of Obstetrics and Gynecology, Reproductive Medicine, Chiba University Graduate School of Medicine, Chiba, Japan; Department of Obstetrics and Gynecology, Chiba University Hospital, Chiba University, Chiba, Japan; Department of Obstetrics and Gynecology, Chiba University Hospital, Chiba University, Chiba, Japan; Department of Obstetrics and Gynecology, Chiba University Hospital, Chiba University, Chiba, Japan; Department of Obstetrics and Gynecology, Chiba University Hospital, Chiba University, Chiba, Japan; Department of Obstetrics and Gynecology, Reproductive Medicine, Chiba University Graduate School of Medicine, Chiba, Japan; Department of Obstetrics and Gynecology, Chiba University Hospital, Chiba University, Chiba, Japan

**Keywords:** false-positive hCG, heterophilic antibody, hydatidiform mole, phantom hCG, trophoblastic disease, urine hCG

## Abstract

Accurately interpreting persistent, low human chorionic gonadotropin (hCG) levels is essential for managing gestational trophoblastic disease. Erroneous interpretation can lead to inappropriate interventions, including unnecessary chemotherapy or hysterectomy, or unjustified changes in chemotherapeutic regimens due to misidentification of a false-positive hCG as a true positive. The predominant etiology of phantom hCG is the presence of heterophilic antibodies. Consequently, screening for urine hCG is indispensable for its diagnosis because immunoglobulin is not generally present in urine. Here, we report about phantom hCG after a complete hydatidiform mole. Initial urine hCG evaluations were negative, although the serum hCG levels remained positive, leading to the diagnosis of phantom hCG. After subsequent delivery, urine hCG levels persisted at diminished levels. However, a different assay yielded negative hCG results for both serum and urine samples. The patient subsequently gave birth. The absence of hCG was consistently confirmed over five years.

## INTRODUCTION

Trophoblastic diseases include hydatidiform moles, invasive moles, choriocarcinomas, placental site trophoblastic tumors, and epithelioid trophoblastic tumors [1]. Invasive moles and choriocarcinomas are categorized as gestational trophoblastic neoplasias (GTNs). Human chorionic gonadotropin (hCG) plays a crucial role in managing gestational trophoblastic disease (GTD) because hCG detection is crucial for GTD diagnosis. GTD remission is determined based on negative hCG findings. Systemic chemotherapy is the standard treatment for trophoblastic tumors when hCG is detected, even if no evaluable lesions are present on imaging [1].

Interpreting persistent low levels of human hCG is important for managing GTD. Misinterpretation can lead to incorrect management, including needless chemotherapy or hysterectomy for molar pregnancy, or unnecessary drug changes during chemotherapy when false-positive hCG might be considered true-positive hCG. Phantom hCG is the cause of needless treatments [2, 3].

The most extensive study available indicated that 73 cases exhibited a negative urinary hCG result while serum hCG tested positive [3]. Most phantom hCG cases are believed to arise from interactions between capture antibodies and luminescent antibodies mediated by heterophilic antibodies. While hCG is excreted in urine, immunoglobulins are typically not excreted via this route. Thus, measuring hCG levels in urine samples is beneficial for distinguishing phantom hCG [2, 3].

Herein, we report about phantom hCG after a complete hydatidiform mole. Finally, the patient did not require chemotherapy, conceived, and had two healthy children.

## CASE REPORT

**Figure 1:**
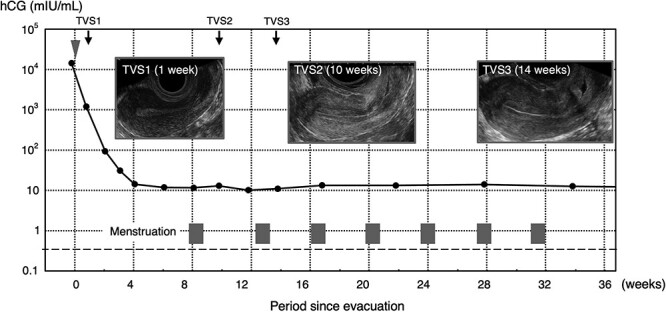
Clinical course of the case. The horizontal axis represents the duration (weeks) after removal of the molar pregnancy, and the vertical axis indicates the hCG level (mIU/ml). The gray triangle denotes the molar pregnancy surgery, and the gray rectangles indicate the menstrual periods. hCG; human chorionic gonadotropin, TVS; transvaginal ultrasonography.

A healthy 23-year-old woman in her previously uncomplicated first pregnancy was referred to our hospital from an office gynecologic clinic at 7 weeks of gestation for a suspected hydatidiform mole. Ultrasound detected a molar pregnancy in the uterine cavity. The hCG level was 14 332 mIU/ml. The pregnancy was evacuated using a vacuum instrument. The pathological diagnosis of the intrauterine content was complete hydatidiform mole. Immunostaining for p57KIP2 was negative in both cytotrophoblasts and villous stromal cells. Subsequently, weekly hCG monitoring was continued (Fig. 1). In the fourth week after evacuation, the hCG level reached 14.2 mIU/mL (IMMULITE 2000 HCG; Siemens Healthcare Diagnostics Inc., Erlangen, Germany). However, hCG levels plateaued six weeks after evacuation (Fig. 1). Menstruation began again eight weeks after evacuation. Transvaginal ultrasonography revealed no foci in the uterus. We measured follicle-stimulating hormone (FSH) and luteinizing hormone (LH) levels to exclude hypothalamic hCG. FSH and LH levels were 5.0 mIU/ml and 8.8 mIU/ml (late follicular phase), respectively. The urine hCG level was below the detection limit (IMMULITE 2000 HCG). In the 22^nd^ week, hCG levels were measured using another kit (E test TOSOH II; TOSOH CORPORATION, Tokyo, Japan). Serum hCG levels were below the detection limit. Finally, in the 34^th^ week (Fig. 1), the serum hCG level of IMMULITE 2000 was still positive at 12.6, although serum and urine hCG of TOSOH II and the urine hCG of IMMULITE 2000 were negative. Thus, we diagnosed the serum low-level persistent hCG of the IMMULITE 2000 as phantom hCG and allowed the next pregnancy. She conceived immediately and delivered her first healthy baby at 39 weeks. Three months after birth, the hCG level in IMMULITE 2000 remained positive. Urine hCG was rarely detected in IMMULITE 2000 (Table 1) for one year. No proteinuria was observed during this period. In contrast, serum and urine hCG levels in the E test TOSOH II were consistently negative, except at one point. The laboratory instruments for hCG measurement at our hospital were changed accordingly. hCG levels were measured using the Elecsys HCG + β (Roche Diagnostics K. K., Tokyo, Japan) kit. She delivered a second healthy baby at 39 weeks. She continued in remission and has not shown new gestational trophoblastic neoplasia for five years.

**Table 1:** Trend of serum and urine hCG levels in different hCG kits

Time after evacuation	IMMULITE 2000 HCG (mIU/ml)		E test TOSOH II (mIU/ml)		Elecsys HCG + βb (mIU/ml)		LH (mIU/ml)	FSH (mIU/ml)
	Serum	Urine	Serum	Urine	Serum	Urine	Serum	Serum
14 w	11	< 1.0					8.8	5
17 w	13.3	< 1.0						
22 w	13.2	< 1.0	< 0.5					
28 w	14.0	< 1.0	< 0.5					
34 w	12.6	< 1.0	< 0.5	< 0.5				
17 m (First delivery)								
20 m	8.1	1.5	< 0.5	< 0.5				
23 m	8.8	1.2	< 0.5	< 0.5				
25 m	5.4	< 1.0	< 0.5					
28 m	6.1	1.6	0.8	< 0.5			7.6	4.9
30 m	6	2.1	< 0.5	< 0.5				
33 m	6.5	1.6	< 0.5	< 0.5				
39 m					< 0.2	< 0.2		
43 m					< 0.2	< 0.2		
49 m					< 0.2			
55 m					2.8		1.4	2.1
61 m					< 0.2			
71 m (Second delivery)								
74 m					< 0.2			

w, weeks; m, months.

IMMULITE 2000 HCG (detection limit: 1.0 mIU/ml, Siemens Healthcare Diagnostics Inc., Erlangen, Germany, immobilized antibody; mouse monoclonal, enzyme-linked antibody; sheep polyclonal).

E test TOSOH II (detection limit: 0.5 mIU/ml, TOSOH CORPORATION, Tokyo, Japan, immobilized antibody; mouse monoclonal, enzyme-linked antibody; mouse monoclonal).

Elecsys HCG + β (detection limit: 0.2 mIU/ml, Roche Diagnostics K. K., Tokyo, Japan, immobilized antibody; mouse monoclonal, enzyme-linked antibody; mouse monoclonal).

## DISCUSSION

The low hCG level in this case was false positive; therefore, we could avoid treatment with chemotherapy for GTN. Many causes of false-positive hCG results have been reported, including pituitary hCG released during menopause, ovarian failure, and heterophilic antibody false positives (human anti-mouse antibodies/human anti-rabbit antibodies) [1]. Non-gestational tumors (germ cells, epithelial cells, or other carcinomas), familial increased hCG [4–7], and injected hCG (infertility treatment or body-building program) [8] result in false positives. The possibility of an unrecognized pregnancy should always be considered. In our case, detecting serum hCG 55 months after the evacuating a molar pregnancy was likely due to a chemical abortion.

The mechanism underlying false-positive hCG detection is considered a heterophilic antibody that can bind to two hCG-specific antibodies for sandwich ELISA. This misjudgment can result in patients with GTN undergoing unnecessary chemotherapy or total hysterectomy. Among 83 false-positive cases, 62 received unwarranted chemotherapy or hysterectomy because of the presumed GTD in a study [3]. It is crucial to measure urine hCG because the heterophilic antibody (IgG) is absent in urine owing to its molecular size [1].

This case suggests that urine hCG is not always accurate for diagnosing phantom hCG in a very small percentage of cases. After the initial delivery and during the 20- to 33-month interval after evacuating the molar pregnancy, urine hCG manifested positive values, albeit at diminished levels. We postulate that identifying a minimal hCG level in the urine might be attributed to heterophilic antibody leakage or secretion into the urine. Urine hCG detection does not guarantee the presence of true hCG.

We have experienced approximately 1200 cases of GTD, including hydatidiform moles and GTN, over 20 years. Almost all patients were followed up until hCG levels were below the detection limit. Specifically, we observed about 1200 cases of low hCG levels. Among them, only one case was diagnosed as ‘phantom hCG,’ suggesting that the frequency of phantom hCG is about 0.1% (1/1200) in the Japanese population. Although the precise prevalence of phantom hCG remains unclear, it is postulated to be notably rare. Given its rarity and that it may seem paradoxical, ensuring that true hCG is not inadvertently overlooked in managing trophoblastic diseases is paramount.
